# *In Vivo* Confocal Microscopy Evaluation of Ocular Surface with Graft-Versus-Host Disease-Related Dry Eye Disease

**DOI:** 10.1038/s41598-017-10237-w

**Published:** 2017-09-06

**Authors:** Jingliang He, Yoko Ogawa, Shin Mukai, Yumiko Saijo-Ban, Mizuka Kamoi, Miki Uchino, Mio Yamane, Nobuhiro Ozawa, Masaki Fukui, Takehiko Mori, Shinichiro Okamoto, Kazuo Tsubota

**Affiliations:** 1 Aier School of Ophthalmology, Central South University, Changsha, China; 2 Department of Ophthalmology, Keio University School of Medicine, Tokyo, Japan; 3 Division of Hematology, Department of Medicine, Keio University School of Medicine, Tokyo, Japan

## Abstract

Dry eye disease (DED) is often elicited by graft-versus-host disease (GVHD), an extensive complication of hematopoietic stem cell transplantation (HSCT). To unravel the mechanism of this type of DED, *in vivo* confocal microscopy (IVCM) was used to investigate alterations in the state of the sub-basal nerves, dendritic cells (DCs) and globular immune cells (GICs) in the central cornea and limbal epithelia. In this study, we examined 12 HSCT recipients with GVHD-caused DED and 10 HSCT recipients without GVHD-associated DED and evaluated the clinical parameters in the 2 groups. Analysis of the central cornea and limbal epithelia using IVCM was conducted to investigate the density of the corneal sub-basal nerves, DCs and GICs as well as the tortuosity and branching of the sub-basal nerves. As suggested by our data, the clinical variables in the GVHD group were significantly different from those in the non-GVHD group. Additionally, GVHD-triggered DED conceivably increased the density of DCs and GICs in the central cornea and the density of DCs in limbal epithelia and altered the morphology of the sub-basal nerves. These phenomena are presumably correlated with the degree of inflammation. Thus, our findings may be translated into non-invasive diagnostic methods that indicate the severity of inflammation on the ocular surface in HSCT recipients.

## Introduction

Hematopoietic stem cell transplantation (HSCT) is widely used for the treatment of hematological malignancies and several benign hematopoietic diseases. Technology has progressed considerably during the last decades, and consequently, the success rate of HSCT itself has substantially improved^1, 2^. However, medical settings are still required to combat disabling complications resulting from HSCT. One of the most notorious disorders is graft-versus-host disease (GVHD), which can affect multiple organs. GVHD is one of the leading causes of morbidity after HSCT and occurs in 10–90% of HSCT recipients^3, 4^. The eye is one of the most GVHD-vulnerable organs, and 40–60% of patients who undergo HSCT develop ocular GVHD^3^. In many cases, patients with ocular GVHD suffer from ocular disorders, visual deterioration, lacrimal gland dysfunction, meibomian gland dysfunction, corneal epitheliopathy, conjunctival scarring, and hyperemia. These conditions induced by ocular GVHD have a large detrimental impact on patients’ quality of life^5, 6^.

Although several effective medications have emerged^7, 8^, the exact pathogenesis of ocular GVHD remains to be elucidated. Previous studies suggest that donor-derived immunocompetent cells attack host tissues and presumably play a contributory role in the development of GVHD^9, 10^. Because there are limited data evaluating the ocular surface in GVHD patients, an investigation into GVHD-elicited changes of the ocular surface at the cellular level could provide some insights into the developmental process that leads to ocular GVHD.

*In vivo* confocal microscopy (IVCM) provides high-resolution images in a non-invasive manner and enables an examination of the ocular surface at the cellular level. In many previous studies, ICVM has been used to assess cellular and structural changes of the ocular surface in patients with various diseases, such as herpes simplex virus (HSV) keratitis, corneal graft rejection, rosacea and dry eye disease (DED), especially chronic GVHD-related dry eye disease^11–14^. GVHD-induced changes in the state and/or behavior of corneal sub-basal nerves, corneal epithelial dendritic cells (DCs), and conjunctival epithelial immune cells have been reported^14^. In this study, the status of the ocular surface in HSCT recipients with GVHD-induced DED was compared with those without DED. Considering that GVHD patients undergo total body irradiation (TBI), usually receive immunosuppressive therapies and have underlying hematopoietic diseases, alterations in the condition of the ocular surface may be induced by these factors^3, 15^. This clinical study comprehensively focused on these matters. Consequently, the results may potentially elucidate the link between GVHD-triggered DED and the deterioration in the state of the ocular surface in HSCT recipients.

## Results

Twenty-two eyes from 22 HSCT recipients (10 males and 12 females) with GVHD-related DED (n = 12) and without GVHD-related DED (n = 10) were examined. The patients’ characteristics in both groups are shown in Table 1. With regard to the types of HSCT, the subjects in the GVHD group underwent bone marrow transplantation (BMT, n = 8) or peripheral stem cell transplantation (PBSCT, n = 4), and those in the non-GVHD group received BMT (n = 5), PBSCT (n = 1) or umbilical cord blood transplantation (CBT, n = 4).

**Table 1:** Patient characteristics.

Characteristics	GVHD (n = 12)	Non-GVHD (n = 10)	P Value
Age, y	52.16 ± 12.55	47.30 ± 18.55	0.473
Gender, no. (male/female)	5/7	5/5	1.000
Time-period after HSCT, m	84.58 ± 65.13	72.60 ± 59.59	0.660
Type of HSCT, no. (BMT/PBSCT/CBT)	8/4/0	5/1/4	
Type of donor, no. (related/unrelated)	5/7	5/5	1.000
Primary disease (no.)			
	AML (4)	AML (3)	
	MDS (4)	HL (1)	
	ALL (2)	ALL (2)	
	CML (1)	CML (1)	
	PTCL (1)	CMML (1)	
		AA (1)	
		NHL (1)	
TBI, no. (yes/no)	8/4	10/0	0.096
Duration of DED, m	67.00 ± 61.74	—	
SIT, no. (yes/no)	7/5	2/8	0.099

BMT: bone marrow transplantation; PBSCT: peripheral blood stem cell transplantation; CBT: umbilical cord blood transplantation; TBI: total body irradiation; SIT: systemic immunosuppressive treatment; AML: acute myeloid leukemia; MDS: myelodysplastic syndrome; ALL: acute lymphocytic leukemia; CML: chronic myeloid leukemia; PTCL: peripheral T-cell lymphoma; HL: Hodgkin lymphoma; CMML: chronic myelomonocytic leukemia; AA: aplastic anemia; NHL: non-Hodgkin lymphoma.

Primary hematological diseases in the GVHD group were acute myeloid leukemia (AML, n = 4), myelodysplastic syndrome (MDS, n = 4), acute lymphocytic leukemia (ALL, n = 2), chronic myeloid leukemia (CML, n = 1) and peripheral T-cell lymphoma (PTCL, n = 1), and those in the non-GVHD group were AML (n = 3), Hodgkin lymphoma (HL, n = 1), CML (n = 1), ALL (n = 2), chronic myelomonocytic leukemia (CMML, n = 1), aplastic anemia (AA, n = 1) and non-Hodgkin lymphoma (NHL, n = 1). There was no significant difference in age range (p = 0.473), gender diversity (p = 1.000), time-period after HSCT (p = 0.660), donor type (p = 1.000), systemic immunosuppressive treatment (SIT, p = 0.099) or ratio of TBI (p = 0.096) between the GVHD and non-GVHD groups. The topical medications used by the patients are shown in Table 2. Four patients in the control group, whose ocular symptoms and signs were minimal, were medicated with sodium hyaluronate. However, these signs and symptoms did not arise from DED; they were induced by other factors such as tiny mechanical damage. Sodium hyaluronate was prescribed to protect the mucosa of the ocular surface and completely relieved the symptoms in these patients.

**Table 2:** Topical medication of patients.

Patient	Topical medication (within 1 month)
G1	Sodium hyaluronate, Diquafosol tetrasodium
G2	Cyclosporine, Diquafosol tetrasodium, Vitamin A
G3	Sodium hyaluronate, Artificial tear, Cyclosporine, Diquafosol tetrasodium
G4	Rebamipide
G5	Sodium hyaluronate, Diquafosol tetrasodium, Rebamipide
G6	Sodium hyaluronate, Artificial tear, Rebamipide, Methylpredonisolon
G7	Sodium hyaluronate, Artificial tear, Rebamipide, Diquafosol tetrasodium
G8	Sodium hyaluronate, Epinastine, Rebamipide, Levofloxacin
G9	Sodium hyaluronate, Rebamipide, Artificial tear, Ofloxacin
G10	Sodium hyaluronate, FK-506, Ofloxacin
G11	Diquafosol tetrasodium
G12	Sodium hyaluronate, brinzolamide ophthalmic suspension
C1	Sodium hyaluronate
C2	none
C3	none
C4	none
C5	none
C6	Sodium hyaluronate
C7	Epinastine, Methylpredonisolon
C8	none
C9	Sodium hyaluronate; Epinastine
C10	Sodium hyaluronate

G: GVHD, C: non-GVHD.

**Figure 1:**
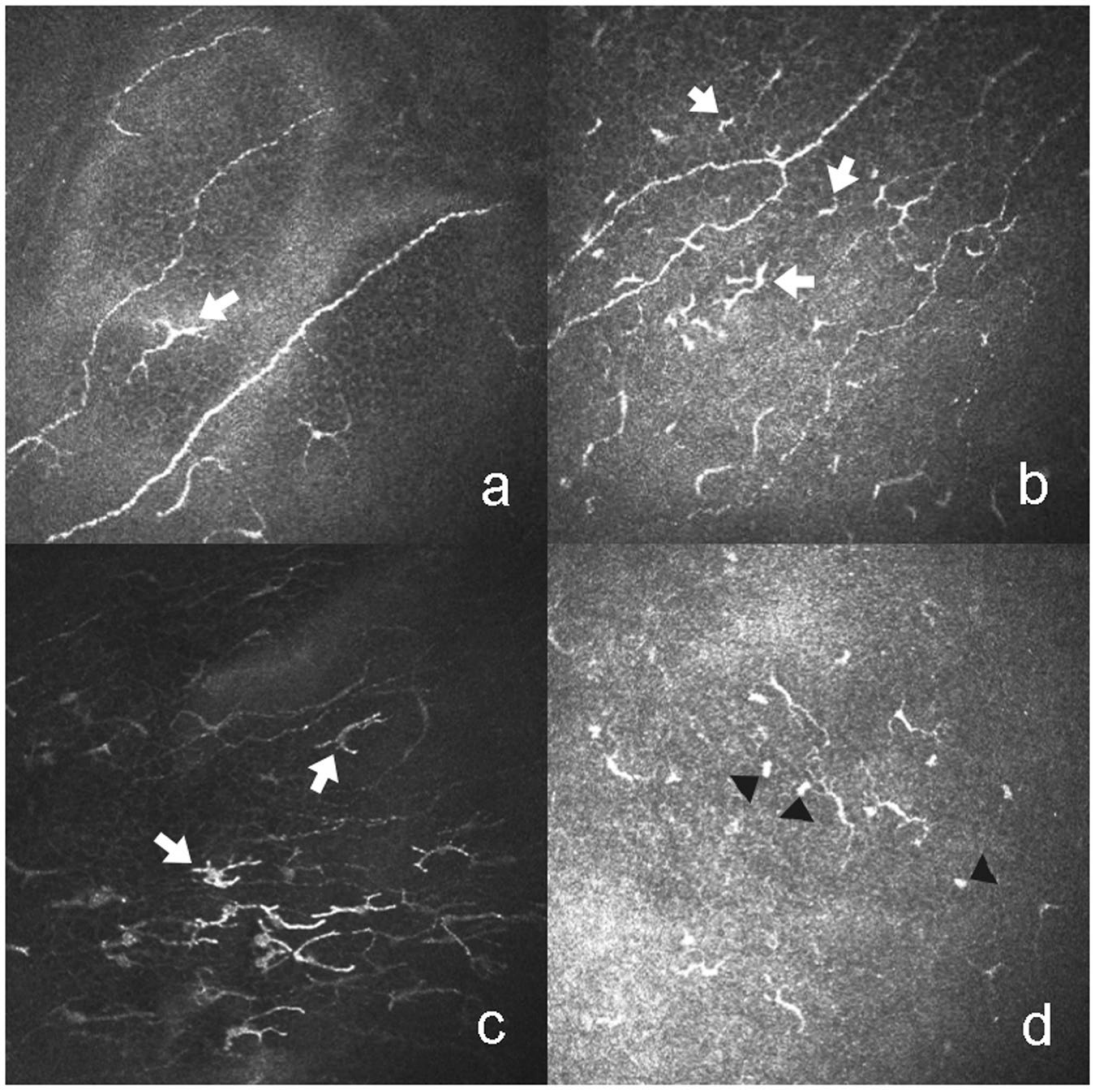
Representative IVCM images of dendritic cells (DCs) and globular immune cells (GICs). (**a**) DCs with typical branching dendrites in the basal layer of the epithelia in the central cornea (white arrow). (**b**) DCs with small cell bodies and short dendrites in basal membranes in the central cornea (white arrow). (**c**) DCs with highly reflective cell bodies and branching dendrites in the basal layer in the limbus (white arrow). (**d**) GICs co-exist with DCs in the layer of the basal membrane in central cornea (black arrowhead).

**Figure 2:**
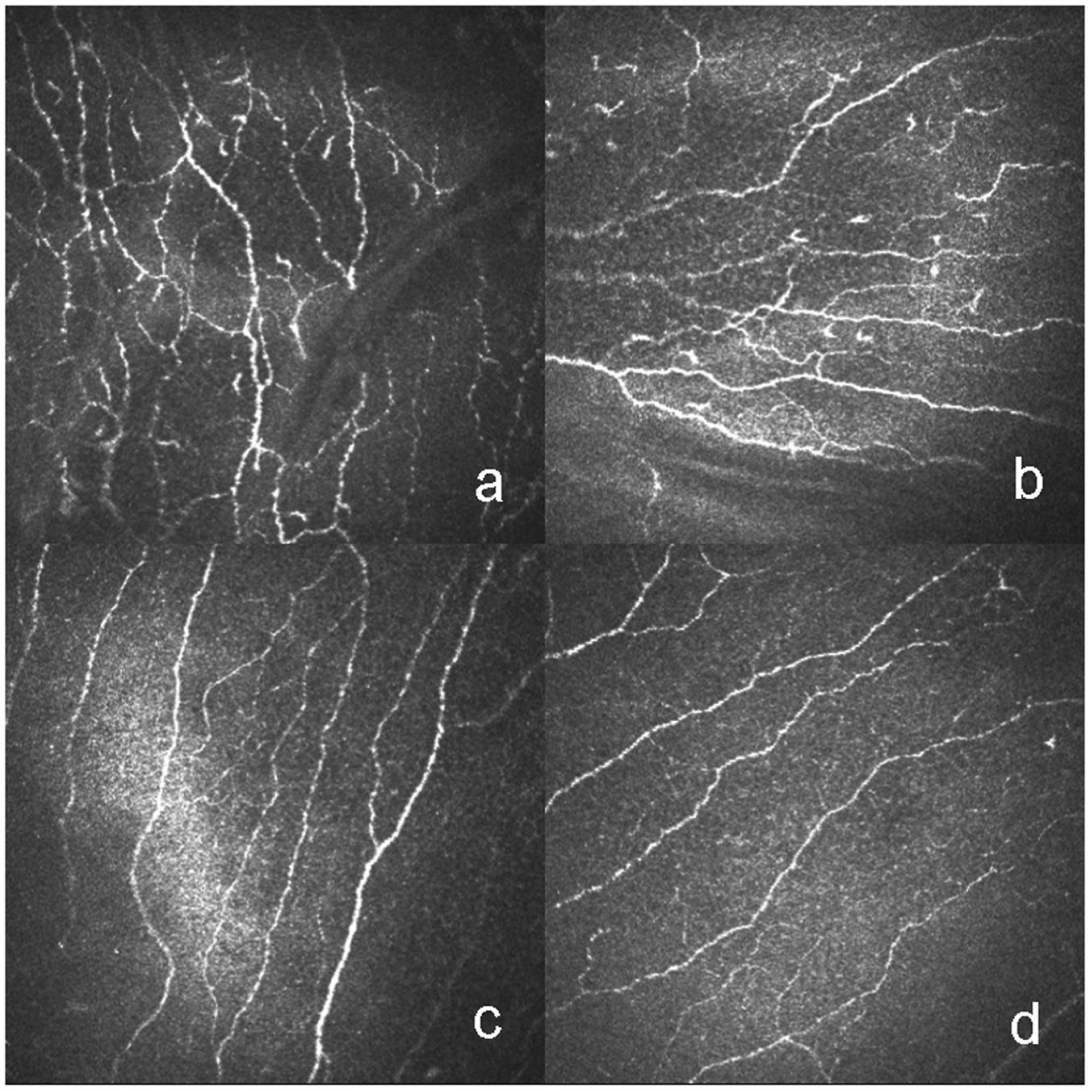
Representative IVCM images of sub-basal nerves in HSCT recipients with GVHD (**a,b**) and HSCT recipients without GVHD (**c,d**). The level of tortuosity and branching, and density of sub-basal nerves are shown in HSCT recipients with GVHD (**a,b**) in comparison with HSCT recipients without GVHD (**c,d**).

**Figure 3:**
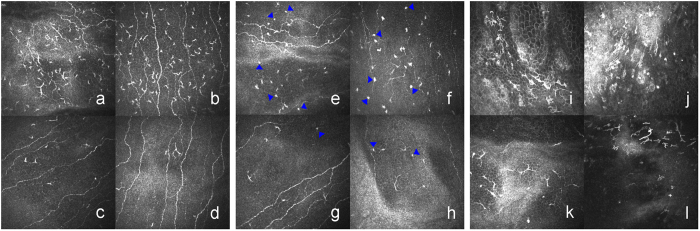
Representative IVCM images of DCs (left panel, a–d) and GICs (middle panel, e–h) in the central cornea and limbal epithelium (right panel, i–l) in GVHD patients (upper raw, **a,b,e,f,i,j**) and non-GVHD patients (lower raw, **c,d,g,h,k,l**). In the central cornea, the density of epithelial DCs in GVHD patients (**a**,**b**) and in non-GVHD patients (**c,d**). The density of GICs (blue arrowhead) in the GVHD group (**e,f**) and in the non-GVHD (**g,h**). In limbal epithelia, the density of DCs in GVHD patients (**i,j**) and non-GVHD patients (**k,l**).

Clinical variables and IVCM parameters are depicted in Table 3. The clinical variables, namely, ocular surface disease index (OSDI), meibomian gland dysfunction score (MGDs), cornea fluorescein staining (CFS), conjunctival lissamine green staining (CLGS) and hyperemia, in the GVHD group were significantly higher than those in the non-GVHD group. Both the Japanese dry eye score (JDEs) and the international chronic ocular GVHD scores (ICOGs) in the GVHD group were significantly higher than those in the non-GVHD group. These latter two parameters were significantly associated with all IVCM parameters except the density of the sub-basal nerves. In contrast, the opposite was seen with Schirmer’s test and tear film break-up time (BUT). As for the results of the IVCM images, DCs with typical branching dendrites in the basal layer of the epithelia in the central cornea were observed in chronic GVHD (cGVHD)-related DED patients (Fig. 1a). In addition, DCs with small cell bodies and short dendrites in the basal membrane in the central cornea were identified (Fig. 1b). DCs with highly reflective cell bodies and branching dendrites in the basal layer in the limbus were also apparent (Fig. 1c). Moreover, globular immune cells (GICs) co-existed with DCs in the basal membrane layer in the central cornea (Fig. 1d). The GVHD group displayed a significantly higher level of tortuosity (Fig. 2a,b) and branching of the sub-basal nerves (Fig. 2a,b) compared with the non-GVHD group (Fig. 2c,d). The density of GICs in the central cornea (Fig. 3e,f), the density of DCs in both the central cornea (Fig. 3a,b) and the limbus (Fig. 3i,j) were significantly higher in the GVHD group compared with the non-GVHD group (Fig. 3c,d,g,h,k,l). Significant correlations were mainly noticed between dry eye parameters and IVCM variables, including the density of DCs and GICs in the central cornea, the density of DCs in the limbal epithelia, and tortuosity and branching of the sub-basal nerve. Detailed correlations between IVCM variables and dry eye parameters are shown in Table 4. These results suggest that more severe infiltration of DCs and GICs on the ocular surface occurs in GVHD patients compared with non-GVHD patients. Furthermore, significant alterations in corneal innervation occur in GVHD-affected eyes. In addition, these IVCM parameters were strongly associated with a variety of dry eye parameters.

**Table 3:** Clinical variables and IVCM parameters.

Parameters	GVHD (n = 12)	Non-GVHD (n = 10)	P Value
OSDI (scores)	44.01 ± 22.66	7.91 ± 9.41	<0.001***
Schirmer’s test (mm)	4.33 ± 6.17	11.10 ± 7.02	0.007**
BUT (s)	2.58 ± 0.67	8.50 ± 2.01	<0.001***
MGDs (scores)	2.08 ± 1.00	0.40 ± 0.84	0.001**
CFS (points)	5.17 ± 2.29	0.20 ± 0.63	<0.001***
CLGS (points)	5.92 ± 1.83	0.40 ± 0.70	<0.001***
Hyperemia (scores)	1.50 ± 0.80	0.10 ± 0.32	<0.001***
JDEs (scores)	1.92 ± 0.29	0.20 ± 0.42	<0.001***
ICOGs (scores)	1.83 ± 0.39	0	<0.001***
Density of DCs in central cornea (cells/mm^2^)	119.29 ± 79.78	37.57 ± 21.72	0.001**
Density of DCs in limbal epithelia (cells/mm^2^)	116.50 ± 52.61	55.57 ± 34.24	0.005**
Density of GICs in central cornea (cells/mm^2^)	41.62 ± 10.05	24.69 ± 25.61	0.003**
Density of sub-basal nerve (mm/mm^2^)	17.80 ± 6.17	13.69 ± 5.08	0.108
Density of nerve branching points (no./mm^2^)	93.97 ± 51.92	46.76 ± 36.57	0.025*
Tortuosity of sub-basal nerve (scores)	3.13 ± 0.68	2.15 ± 0.50	0.001**

*p < 0.05; **p < 0.01; ***p < 0.001. OSDI: ocular surface disease index; BUT: tear film break-up time, CFS: cornea fluorescein staining; CLGS: conjunctival lissamine green staining; MGDs: meibomian gland dysfunction score; JDEs: Japanese dry eye score; ICOGs: International chronic ocular GVHD score; DCs: dendritic cells; GICs: globular immune cells.

**Table 4:** Correlations between IVCM parameters and clinical variables.

	OSDI	Schirmer’s test	BUT	MGDs	CFS	CLGS	JDEs	ICOGs
**Density of DCs in central cornea**								
r	0.670	−0.585	−0.491	0.461	0.569	0.597	0.749	0.729
p	0.001**	0.004**	0.002**	0.031*	0.006**	0.003**	0.000***	0.000***
**Density of DCs in limbal epithelia**								
r	0.369	−0.276	−0.732	0.304	0.520	0.678	0.596	0.586
p	0.091	0.214	0.000***	0.169	0.013*	0.001**	0.003**	0.004**
**Density of GICs in central cornea**								
r	0.525	−0.362	−0.505	0.620	0.596	0.591	0.686	0.639
p	0.012*	0.098	0.017*	0.002**	0.003**	0.004**	0.000***	0.001**
**Density of sub-basal nerve**								
r	0.196	−0.303	−0.220	−0.045	0.178	0.473	0.281	0.413
p	0.383	0.171	0.325	0.842	0.429	0.026*	0.206	0.056
**Density of nerve branching**								
r	0.361	−0.526	−0.384	0.029	0.418	0.587	0.444	0.511
p	0.098	0.012*	0.077	0.898	0.053	0.004**	0.038*	0.015*
**Tortuosity of sub-basal nerve**								
r	0.610	−0.422	−0.594	0.264	0.530	0.692	0.591	0.603
p	0.003**	0.050	0.004**	0.235	0.011*	0.000***	0.004**	0.003**

*p < 0.05; **p < 0.01; ***p < 0.001. r: correlation coefficient.

## Discussion

Corneal innervation is one of the most important aspects indicating the state of the ocular surface. As evidenced by previous studies using IVCM, various diseases can alter the density, tortuosity and branching of corneal nerves. As indicated by our study, the density of sub-basal nerves in both GVHD and non-GVHD groups was lower than that in normal eyes (21.6–25.9 mm/mm^2^)^16–20^. Kheirkhah *et al*.^13^ reported that the density of sub-basal nerves in patients with GVHD-caused DED was 16.3 ± 6.1 mm/mm^2^, a value similar to ours and that supports the accuracy of our data. Our literature review revealed that the density of the sub-basal corneal nerve is thought to be positively correlated with the corneal sensitivity^21, 22^. Indeed, our earlier work also demonstrated (1) a decrease in corneal sensitivity in HSCT recipients with and without ocular GVHD-induced DED and (2) that there was no significant difference in corneal sensitivity between the two groups^23^. Our new data indicating the decreased density of sub-basal corneal nerves in both GVHD and non-GVHD groups is therefore consistent with that presented in our previous study. Based on these observations, the conditioning regimen that HSCT recipients undergo may well be one of the factors that decreases the sub-basal nerve density. Other research groups have reported that the nerve density in patients with and without Sjögren’s syndrome (SS) is not only decreased due to DED^22, 24, 25^ but is also affected by other autoimmune diseases such as rheumatic arthritis, which is not associated with SS^26^. However, we found no significant difference in sub-basal nerve density between the GVHD and non-GVHD groups.

Our results also indicated that the density of sub-basal nerves could also be altered by the conditioning regimen HSCT patients receive, and not only by DED-related factors and GVHD-elicited aberrant immune responses. It is conceivable that the changes in sub-basal nerve density caused by GVHD-related DED are overshadowed by other co-existing conditions. However, several other studies found no difference in sub-basal nerve function between DED patients and control subjects^27, 28^. These contradictory claims about the density of sub-basal nerves are presumed to arise from the difference in the severity and/or stage of DED. Hence, this discrepancy may partly explain why the density of sub-basal nerves in the GVHD group was not significantly different from that in the non-GVHD groups.

We also found that the tortuosity of sub-basal nerves in the GVHD group was considerably higher compared with that in the non-GVHD group. Steger *et al*.^29^ also reported that the tortuosity of sub-basal nerves in patients with cGVHD-related severe DED was vastly greater than that in 6 patients without these conditions. Our result is consistent with those reported by other groups that have undertaken DED studies^25, 30–33^. In addition, earlier work suggested that the tortuosity of sub-basal nerves was increased in autoimmune diseases such as Grave’s orbitopathy^34^ and rheumatic arthritis^26^.

In addition to the tortuosity, we investigated the branching of sub-basal nerves. Interestingly, our observations suggested that the sub-basal nerves in the GVHD group were branched at a greater level compared with those in the non-GVHD group, while Steger and colleagues^29^ reported that cGVHD-related severe DED decreased the number of branched nerves. There are inconsistencies across several published reports regarding DED-induced alterations in nerve branching. Zhang and colleagues^30^ reported that basal nerves were substantially more likely to branch in patients with Sjögren’s syndrome-associated aqueous tear deficiency (SS-ATD) compared with their counterparts without these conditions. In contrast, another study suggested that the degree of nerve branching in DED patients was lower in comparison to that in normal subjects^22^. Lastly, several groups reported no significant difference in nerve branching between patients with DED and normal control subjects^24, 25^.

The increased tortuosity and branching of sub-basal corneal nerves are thought to result from nerve regeneration caused by the simultaneous action of damaging phenomena and nerve growth factors (NGF) and/or cytokines secreted on the ocular surface during the inflammatory process^30, 31^. Thus, the difference in the stage and severity of DED and the degree of localized inflammation caused by DED presumably account for the inconsistent findings of several research groups about changes in the sub-basal nerves. There is sparse information about the exact mechanisms underlying the morphological changes in sub-basal nerves induced by ocular surface diseases. Furthermore, it is unknown whether the alterations are linked with clinical signs and symptoms of ocular surface disorders. However, our study has provided a clue to solve these riddles. The tortuosity of sub-basal nerves and the level of nerve branching appeared to be increased in the GVHD group. It is therefore conceivable that GVHD-related DED induces morphological alterations in the sub-basal nerves, which promotes severe inflammation on the ocular surface in HSCT recipients. The degree of the morphological changes is presumably an indication of the extent of inflammation that has occurred on the ocular surface.

DCs, which are also referred to as Langerhans cells (LGs) in the epidermis and mucosal membranes of the ocular surface, act as main APCs on the ocular surface and play a vital role in maintaining immune homeostasis in the eyes. Additionally, they are known as key players in the initiation of immune responses^35–38^. Therefore, we evaluated the behaviour of DCs in the central cornea and limbal epithelia in HSCT recipients with or without GVHD-related DED using IVCM. The literature suggests that IVCM has several advantages that enable uncomplicated detection and quantification of DCs in the central cornea and limbal epithelia^11, 39^. However, IVCM allows us to detect other kinds of cells in addition to DCs and can only provide information on the morphology and location of cells. Therefore, identifying DCs only based on morphology can be scientifically inadequate. Macrophages, for instance, may also have a dendritic shape^40, 41^. However, because they are reported to be found exclusively in the corneal stroma^40, 41^, we therefore identified cells with dendrites in epithelia as DCs.

In this clinical investigation, the central corneas in the GVHD group had a higher density of DCs (119.29 ± 79.78 cells/mm^2^) than those in the non-GVHD group (37.57 ± 21.72 cells/mm^2^). One of the novelties of our clinical study was that we compared the density of DCs in HSCT recipients with and without GVHD. Consistent with our finding, Kheirkhah *et al*. previously reported a similar density of DCs in the central corneas of GVHD patients (146.71 ± 93.36 cells/mm^2^)^14^, supporting the accuracy and dependability of our clinical examination. Our results also indicated that the density of DCs in the limbal epithelia in HSCT recipients with GVHD was considerably greater than that in those without GVHD (116.50 ± 52.61 vs. 55.57 ± 34.24 cells/mm^2^). These outcomes are presumably indicative of the higher degree of immune activation and inflammation on the ocular surface in GVHD patients and suggest that the changes in DCs in the central cornea and limbus in HSCT recipients with DED are induced by ocular GVHD instead of the conditioning regimen prior to HSCT.

According to recent studies, DED is presumed to be one of the factors that can increase the density of DCs in corneal epithelia^12, 42, 43^. In regard to immune-mediated diseases, several pieces of evidence suggest that the density of corneal epithelial DCs is augmented in patients with autoimmune diseases such as Steven-Johnson syndrome^44^, rheumatoid arthritis^26, 45^, systemic lupus erythematosus^46^, and ankylosing spondylitis^47^. Therefore, it is plausible that the interaction between immune cells causes many inflammatory cells to infiltrate into the corneal epithelia affected by cGVHD. Furthermore, our literature search revealed that the density of corneal DCs in DED patients with SS^33, 42, 48, 49^ and GVHD^12^ is higher than that in DED patients without these immunological disorders. The development of these immune-mediated diseases is also presumed to contribute detrimentally to the changes in the behaviour of DCs in the corneal epithelia. However, Dana and colleagues reported different results. They recruited DED patients with chronic GVHD (n = 33) and DED patients who did not receive HSCT and were therefore without chronic GVHD (n = 21). Then, they compared IVCM variables in the two groups. As the clinical severity of DED in the GVHD group was significantly greater than that in the control group, they made adjustments to the clinical severity of DED in order to prevent the difference in the severity of DED between the two groups from affecting IVCM variables. After the adjustments, there was no significant difference in IVCM variables between DED patients with GVHD and those who did not undergo HSCT and were therefore free of GVHD^14^.

Because of the close link between the propagation of GVHD and the progression of DED, a painstaking investigation will be required to distinguish the separate effects of these two events on the changes observed using IVCM. However, as indicated by this clinical investigation, the density of corneal epithelial DCs in HSCT recipients with GVHD was greater than those without GVHD, and the IVCM parameters in the cornea were associated with various clinical variables. Therefore, the density of corneal DCs could be a reliable indicator of the severity of ocular surface damage and inflammation in GVHD patients. Additional studies are needed to corroborate this notion.

We also focused on GICs in the basal epithelial and sub-basal layers, which have been presumed to play a role in inflammatory ocular diseases^11^ and DED^42^. The density of GICs in the central cornea in the GVHD group was significantly higher than that in the non-GVHD group. The increased infiltration of GICs may be indicative of a more active immune response. However, it is still unknown why GICs infiltrate into the ocular surface and how they function in that capacity. It is challenging to unravel the phenotype of these types of cells through morphological investigation using IVCM because they do not have characteristic shapes. Continuing endeavours are needed to uncover the phenotype and function of GICs and their role in the pathophysiology of GVHD-related DED.

This clinical study had several limitations. First, the relatively small number of samples in this study did not allow us to comprehensively investigate the difference in IVCM variables between the different severities of GVHD-related DED. We were also unable to subdivide the 2 main groups (GVHD and non-GVHD groups) according to other clinical aspects such as whether HSCT recipients did or did not undergo TBI. Second, although the shapes of DCs presumably reflect the state of activation, maturation and antigen-presenting capability of DCs, we did not explore certain morphological characteristics of DCs, such as their size, the number of dendrites and the dendritic field. More work is required to determine the nature of the correlation between the morphological alterations of DCs and the extent of inflammation induced by GVHD-related DED. In addition, only the inferior limbal epithelia in HSCT recipients with and without GVHD were assessed in this study. Ideally, four quadrants of the limbus should be evaluated in order to comprehensively examine the limbus. However, prolonged examinations are needed to achieve this and would require placing the patients, especially those with GVHD, in very uncomfortable situations. Clinical characteristics such as age range, gender diversity, primary diseases, history of TBI and types of HSCT in the GVHD group were not exactly the same as those in the non-GVHD group. In reality, it is extremely difficult to match the GVHD and non-GVHD groups with respect to the above aspects, since the recruitment of HSCT recipients, especially those without GVHD-related DED, is highly demanding.

In summary, the density of DCs in both the central cornea and limbal epithelia in HSCT recipients with GVHD-related DED was significantly higher compared with those without these conditions. In addition, the sub-basal corneal nerves in HSCT recipients with GVHD-related DED appeared to be more vulnerable to morphological changes in comparison with those in HSCT recipients without GVHD-related DED. All in all, these phenomena discovered through IVCM are presumed to be associated with the status of ocular GVHD-elicited inflammation in the eyes. Our clinical study has the great potential to promote the development of diagnostic technologies to monitor the state of inflammation on the ocular surface in HSCT recipients.

## Methods

### Patients

This study was approved by the Institutional Review Board and Ethics Committee of Keio Hospital (IRB No.: 20130013) and complied with the tenets of the Declaration of Helsinki. The individual participants were notified of all possible consequences of this study, and written informed consent was obtained from each of the subjects.

The GVHD group had 12 HSCT recipients with GVHD-related DED who survived for more than 100 days after the transplantation, and the non-GVHD group had 10 equivalents without GVHD-related DED. The patients were 20 years or older when they were recruited.

The subjects in the GVHD group met the Japanese diagnostic criteria for dry eye. The diagnostic criteria are as follows: (1) disturbance of the tear film (Schirmer test ≤ 5 mm or tear film breakup time ≤ 5 seconds); (2) conjunctivocorneal epithelial damage (fluorescein staining score ≥ 3 points or rose bengal staining score ≥ 3 points); and (3) dry eye symptomatology. Patients were diagnosed with definite dry eye only if they met all 3 diagnostic criteria^50^. Exclusion criteria were: (1) inability to undergo an IVCM examination due to poor physical and/or mental states, (2) written informed consent was not obtained, (3) other ophthalmic diseases such as cataract, retinal detachment and diabetic retinopathy, (4) ocular surgery performed within 6 months prior to the start of this clinical study, (5) current or previous use of contact lenses, (6) signs and symptoms of DED before HSCT, and (7) presence of fundus hemorrhage or uveitis.

In this study, the following data and information were collected from the individual subjects: primary hematological diseases, types of HSCT, TBI history, systemic immunosuppressive therapies, topical medication, post-transplantation period, duration after the onset of GVHD-related DED, OSDI, Schirmer’s test without anesthesia, BUT, CFS, CLGS, MGDs, hyperemia, JDEs^51^ and ICOGs^52^.

### *In vivo* confocal microscopy

Central cornea and inferior limbal epithelia in all patients were examined using *in vivo* confocal laser scanning microscopy (the Rostock Corneal Software, ver. 1.2, Heidelberg Retina Tomograph II [RCM/HRT II]; Heidelberg Engineering GmbH). At the beginning of examination, the proper volume of the topical anesthetic 0.4% oxybuprocaine was administered to the conjunctival sac. A drop of gel (Bausch & Lomb, GmbH, Berlin, Germany), which served as a coupling medium, was placed on the anterior surface of a polymethylmethacrylate (PMMA) cap (Tomo-cap; Heidelberg Engineering GmbH). The individual subjects were instructed to place their heads in a head holder and continue looking forward (during examination of the central cornea) or looking up (during examination of the inferior limbal epithelia). Then, the examiner placed an applanate PMMA cap onto the area of interest, and the state of the area covered by the PMMA cap was examined through real-time video images displayed on a computer screen. The target areas and reference layers were determined by adjusting a controller and a zoom gear. Once all required adjustments were made, the examiner took images by pressing a foot pedal. Under the “sequence model”, this machine provides automatic collection and storage of 100 images (400 × 400 μm in size) per sequence scan. While operating this machine, the examiner adjusted the focal plane to ensure that all target layers of each target area were clearly recorded. To avoid overlap, three non-overlapping scans in each area were performed.

### Image analysis

Three representative images of DCs, GICs and sub-basal nerves in each area were selected for image analysis. These images were all obtained in or behind the basal epithelial layer and in front of Bowman’s layer. All the images obtained were analyzed using ImageJ software (available at: www.imagescience.org/meijering/software). DCs, which have branching dentritic structures, were identified morphologically, and their cellular images were bright (Fig. 1). This description of DCs was presented in a previous publication^11^. GICs possessed round or oval structures with high reflectivity, and their diameter ranged from 5 μm to 15 μm (Fig. 1).

The density of DCs and GICs was defined as the number of cells per mm^2^. The number of cells was counted manually. The analysis of the sub-basal nerves was performed utilizing NeuronJ, a plug-in program for ImageJ, which can semi-automatically trace nerve fibers and measure their total length. The density of sub-basal nerves was defined as the total length of sub-basal nerve fibers per mm^2^. The tortuosity of the sub-basal nerves was graded from 0 to 4, in accordance with a previously published protocol^53^. The branching of the sub-basal nerves was defined as the number of branching points in sub-basal nerves per mm^2^. Three different subareas in each area of interest were photographed and analyzed, and the individual clinical parameters described above were subsequently obtained by averaging the values arising from the analysis. All the images acquired were evaluated by two masked observers (Y.O. and J.H.), and the average of the scores given by the two observers was shown as the value of each image.

### Statistical analysis

Statistical analysis was performed using SPSS software 22 (SPSS, Inc., Chicago, IL, USA). Continuous variables were reported as the mean ± standard deviation (SD). Categorical variables were presented as frequency or percentage. The Shapiro-Wilk test and the Levene test were conducted to investigate the distribution of data and homogeneity of variances in the data, respectively. Student’s t-test was used to compare the means of the data with a normal distribution and homogeneity of variances in the GVHD and non-GVHD groups. The Welch separate variance estimation t-test was conducted for the data with a normal distribution but without homogeneity of variances. The Mann-Whitney U test was used for the comparison of data with skewed distribution. The χ^2^ test was used for the comparison of qualitative variables. A correlation between IVCM variables and clinical variables was assessed using Spearman’s rank-order correlation test. All p values were 2-sided and considered statistically significant when less than 0.05.
